# A method of respiratory phase optimization for better dose sparing of organs at risks: A validation study in patients with lung cancer

**DOI:** 10.18632/oncotarget.23353

**Published:** 2017-12-17

**Authors:** Seong-Hee Kang, Siyong Kim, Dong-Su Kim, Tae-Ho Kim, So-Hyun Park, Dong-Seok Shin, Kyeong-Hyeon Kim, Min-Seok Cho, YeonSil Kim, Tae Suk Suh

**Affiliations:** ^1^ Department of Biomedical Engineering and Research Institute of Biomedical Engineering, The Catholic University of Korea, Seoul, South Korea; ^2^ Department of Radiation Oncology, Virginia Commonwealth University, Virginia, USA; ^3^ Department of Radiation Oncology, College of Medicine, Yonsei University, Seoul, South Korea; ^4^ Department of Radiation Oncology, The Catholic University of Korea, Seoul, South Korea; ^5^ Department of Radiation Oncology, Seoul National University Hospital, Seoul, South Korea

**Keywords:** respiratory gated radiotherapy, breath-hold, lung cancer, respiratory phase optimization, overlap volume histogram

## Abstract

**Background:**

To propose an effective and simple cost value function to determine an optimal respiratory phase for lung treatment using either respiratory gating or breath-hold technique.

**Results:**

The optimized phase was obtained at a phase close to end inhalation in 11 out of 15 patients. For the rest of patients, the optimized phase was obtained at a phase close to end exhalation indicating that optimal phase can be patient specific. The mean doses of the Organs-at-risk (OARs) significantly decreased at the optimized phase without compromising the planning target volume (PTV) coverage (about 8% for all 3 OARs considered).

**Materials and Methods:**

Fifteen lung patients were included for the feasibility test of the cost function. For all patients and all phases, delineation of the target volume and selected OARs such as esophagus, heart, and spinal cord was performed, and then cost values were calculated for all phases. After the breathing phases were ranked according to the cost values obtained, the relationship between score and dose distribution was evaluated by comparing dose volume histogram (DVH).

**Conclusions:**

The proposed cost value function can play an important role in choosing an optimal phase with minimal effort, that is, without actual plan optimization at all phases.

## INTRODUCTION

To acquire maximal benefit with a respiratory gated radiation therapy (RGRT) or breath-hold technique, it is important to properly select patient specific gating window (i.e., breathing phases) [1]. Phases close to end exhalation are usually chosen as gating windows in RGRTs because of longer duration, less tumor motion and better reproducibility [2-4]. There are advantages in end inhalation as well such as lung volume enlargement which may reduce lung complication. For example, there are studies reporting that RGRT has a little benefit on toxicity parameters at end inspiration [5-6]. Breath-hold techniques are often used at end inspiration mainly because of dosimetric benefit with reduced lung density [7]. Deep inspiration breath hold (DIBH) technique, for instance, has been well recognized in terms of the advantage of reduced toxicity to surrounding normal lung tissues [7-10].

Besides the lung itself, there are several other important organs at risk (OARs) such as esophagus, heart and spinal cord in lung cancer treatments. Therefore, it is necessary to consider the movement (and/or deformation) of such OARs as well for treatment planning [11]. Weiss et al. analyzed esophagus and spinal cord motion relative to the gross target volume (GTV) motion using 4-dimensional computed tomography (4DCT), and found that the distance between the GTV and esophagus or spinal cord could change differently phase to phase due to different motion and deformation of each normal structure [12]. Therefore, there may exist an optimal phase that can provide better dose distributions and is not necessarily either end exhalation or end inhalation.

One of the most critical factors that influences dose to OARs surrounding the planning target volume (PTV) is the distances between the PTV and OARs [13]. In other words, the amount of unnecessary dose to the OAR strongly depends on the proximity between the OAR and a target. Since the location of the target and OARs can be different from phase to phase, it is important to properly consider the geometric relationship between the target volume and OARs for all phases. Intuitively, delivered dose to an OAR strongly depends on its proximity to (or overlap with) the PTV [13-15]. Overlap volume histogram (OVH) is an effective descriptor containing the information of the spatial configuration between the target and an OAR [13, 16]. The OVH was successfully applied to intensity modulated radiation therapy (IMRT) planning that generated achievable dose volume histogram (DVH) objectives using previous patients’ information in head & neck and pancreas cases [16-17].

In this study, we introduced a new, simple cost value function that does utilize both the OVH and OAR specific tolerance dose (as biological indicator) to find an optimal phase in dose distribution perspective for lung cancer patients. Validation of the proposed method was performed by comparing dosimetric parameters between respiratory phases for 15 lung cancer patients.

## RESULTS

### Characteristic of cost value function

**Figure 1:**
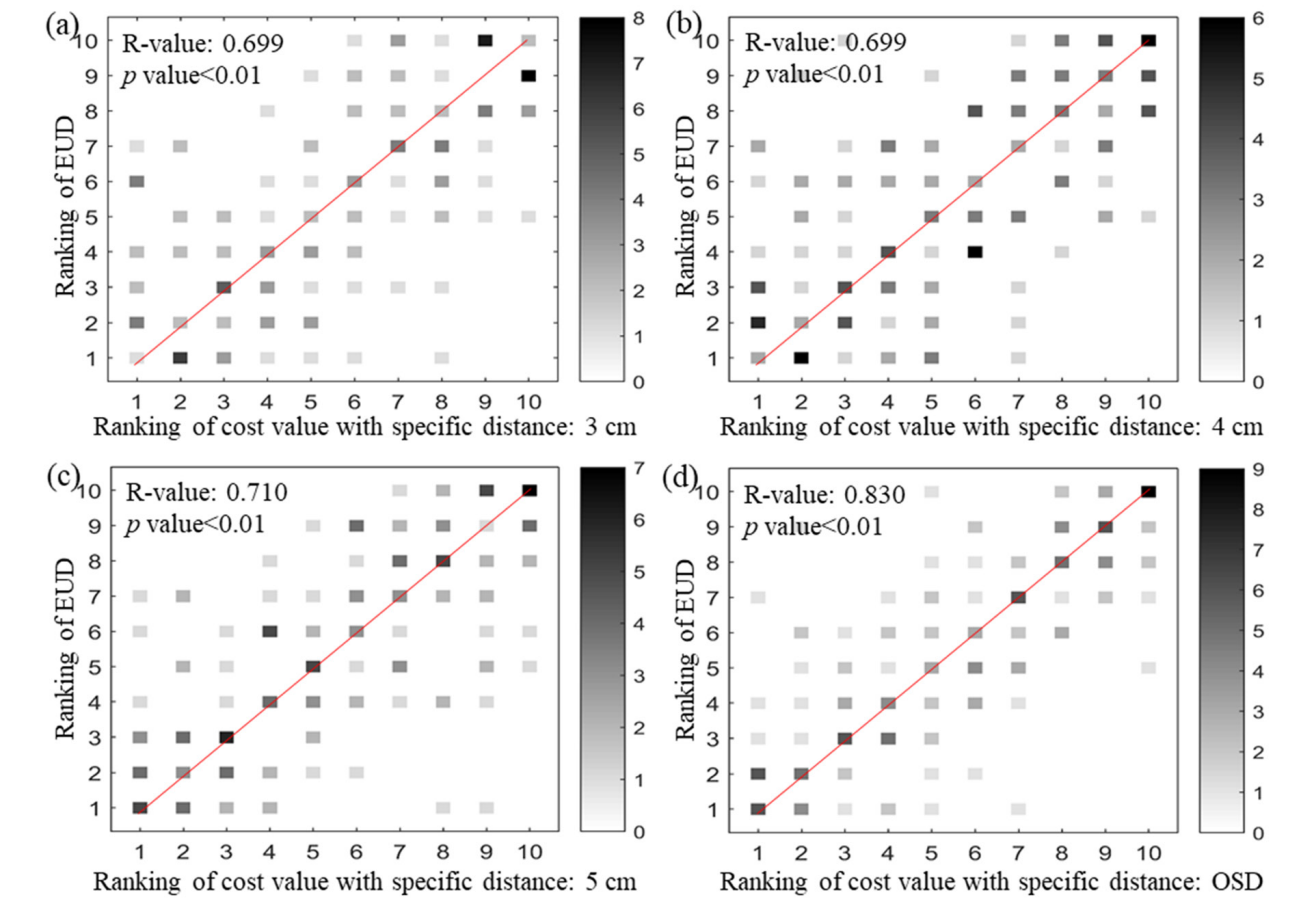
Correlation analysis between ranking of EUD and ranking of the cost value with various specific distances The strongest correlation was obtained when OSD was used as specific distance: (**a**) specific distance = 3 cm; (**b**) specific distance = 4 cm; (**c**) specific distance = 5 cm; (**d**) specific distance = OSD.

Figure 1 shows the correlation analysis between cost value rankings and equivalent uniform dose (EUD) rankings for all patients. It was found that the strongest correlation (R-value: 0.830) was made from the cost values acquired using organ specific distance (OSD) and the weakest correlation (R-value: 0.699) was with 3 cm. Correlation coefficient values are summarized in Table 1. ‘W_*i,d*_ = variable’ and ‘W_*i,d*_ = 1’ indicate that the cost values were obtained with and without distance weighting applied, respectively. We observed that the trend could be well approximated with a linear function with the slope of -14.6% per cm. This slope was used for distance weighting factor calculation. For example, a unit volume would have 14.6% less importance than another unit volume that is located 1 cm closer to the edge of the PTV.

**Table 1:** The correlation coefficient between EUD and cost values with various specific distances.

Patient	r = 3		r = 4		r = 5		OSD	
	*W*_*i,d*_*=1*	*W*_*i,d=*_ variable	*W*_*i,d*_*=1*	*W*_*i,d=*_ variable	*W*_*i,d*_*=1*	*W*_*i,d=*_ variable	*W*_*i,d*_*=1*	*W*_*i,d=*_ variable
1	**0.907**	0.901	**0.894**	0.885	**0.870**	0.863	**0.917**	0.913
2	0.917	**0.934**	0.911	**0.919**	0.892	**0.910**	0.909	**0.918**
3	0.537	**0.537**	0.467	**0.510**	**0.530**	0.467	0.419	**0.426**
4	0.354	**0.355**	0.567	**0.568**	0.537	**0.541**	**0.691**	0.664
5	N/A	N/A	0.286	**0.322**	**0.358**	0.353	0.295	**0.301**
6	**0.610**	0.564	**0.745**	0.739	**0.621**	0.598	**0.789**	0.783
7	0.556	**0.576**	0.462	**0.483**	**0.736**	0.706	0.634	**0.656**
8	**0.562**	0.458	0.576	**0.579**	0.502	**0.514**	0.647	**0.651**
9	0.882	**0.888**	0.910	**0.917**	0.832	**0.842**	0.940	**0.947**
10	0.810	**0.811**	**0.720**	0.615	0.403	**0.451**	**0.822**	0.817
11	**0.688**	0.662	0.706	**0.757**	0.648	**0.716**	0.845	**0.849**
12	0.780	**0.806**	**0.886**	0.853	0.714	**0.816**	0.906	**0.921**
13	**0.623**	0.555	**0.786**	0.642	0.925	**0.932**	0.901	**0.916**
14	**0.774**	0.767	0.810	**0.813**	**0.836**	0.832	0.849	**0.875**
15	**0.927**	0.926	0.930	**0.933**	0.840	**0.844**	0.937	**0.937**

W_i,d=_ variable and W_i,d_=1 indicate the cost values with and without reflecting the distance weighting.

In most cases, higher correlations were observed with the distance weighting applied than those obtained without the distance weighting except for cases with the specific distance *r* = 3 cm. For example, it was possible to obtain a higher correlation when reflecting the distance weighting for cost value calculation under the OSD in 11 out of 15 patients. In patient # 5, no cost value was acquired with the specific distance *r* = 3 cm because there was no overlap volume between the expanded PTVs and OARs at that distance. Among all the cases, the highest correlation was observed in the patient #9 (R value: 0.947), and the lowest correlation in the patient #5 (R value: 0.301) under the OSD with distance weighting.

### Cost value at breathing phase

As shown in Table 2, the lowest cost values were distributed at not a specific breathing phase such as end exhalation or end inhalation but various phases. In 11 out of 15 patients, the lowest cost value was obtained at a phase close to end inhalation (0, 10, 80 or 90% phase). Interestingly, 2 patients (i.e., 2 out of 15 patients), the lowest cost value was obtained at a phase close to end exhalation (30, 40 or 50% phase), indicating that optimal phase can be patient specific as assumed. On the other hand, the highest cost value was observed at end exhalation in 10 out of 15 patients.

**Table 2:** Example of cost values distribution (*r* = OSD) with W_*i,d*_ = variable for each phase; the phase with the lowest cost value (noted in bold) is assumed to be an optimal phase for saving the selected surrounding OARs (*i.e*. esophagus, heart, and spinal cord).

Patient	0%	10%	20%	30%	40%	50%	60%	70%	80%	90%
1	0.0011	0.0015	0.0017	0.0029	*0.0087*	0.0086	0.0076	0.0027	0.0032	***0.0008***
2	0.0098	0.0293	0.0475	0.0400	0.0396	*0.0504*	0.0157	0.0302	0.0337	**0.0092**
3	0.0206	0.0080	0.0058	***0.0039***	0.0042	*0.0408*	0.0163	0.0135	0.0164	0.0140
4	***0.0117***	0.0131	0.0170	0.0178	0.0182	*0.0184*	0.0151	0.0145	0.0153	0.0182
5	***0.0080***	0.0119	0.0231	0.0278	0.0512	*0.0520*	0.0083	0.0146	0.0136	0.0126
6	*0.0283*	0.0210	0.0153	0.0094	0.0075	***0.0036***	0.0107	0.0183	0.0184	0.0181
7	0.0178	***0.0088***	0.0098	0.0089	0.0099	0.0107	0.0136	0.0142	*0.0469*	0.0350
8	0.0144	0.0160	***0.0019***	0.0044	*0.0189*	0.0025	0.0142	0.0137	0.0157	0.0082
9	***0.0084***	0.0185	0.0110	0.0121	0.0181	*0.0297*	0.0236	0.0241	0.0149	0.0102
10	0.0055	0.0065	0.0078	*0.0090*	0.0071	0.0075	0.0070	0.0041	***0.0039***	0.0044
11	0.0290	0.0343	***0.0096***	0.0575	0.0269	0.0542	0.0490	0.0430	0.0501	*0.0812*
12	0.0211	0.0614	*0.1011*	0.037	0.0631	0.0221	0.0908	0.0581	0.0396	***0.0142***
13	***0.0151***	0.0906	0.2423	0.0205	0.1386	*0.3834*	0.3118	0.1811	0.0906	0.049
14	0.0100	0.1829	0.1634	0.0962	*0.1906*	0.0682	0.1680	0.0863	0.1054	***0.0045***
15	0.0055	0.1076	*0.1926*	0.0195	0.1271	0.1100	0.1311	0.1901	0.0066	***0.0018***

### Comparison of dosimetric parameters

**Figure 2:**
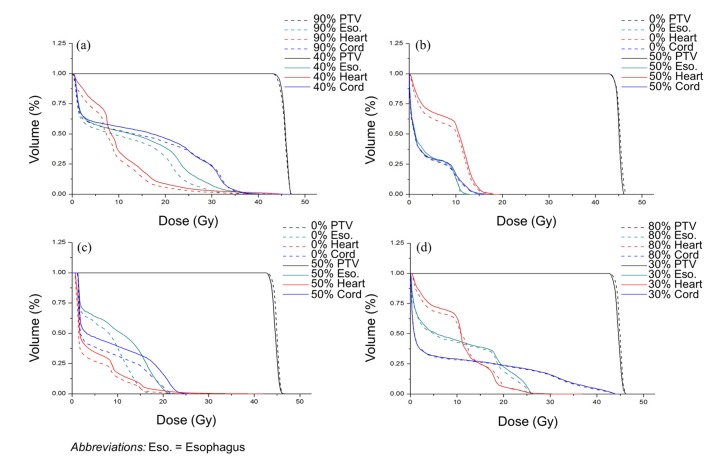
Comparison of the DVH curves between the phase of the lowest cost value and the other at the highest The dose to esophagus, heart, and spinal cord is considerably decreased without compromising PTV coverage at the phase with lowest cost value. (**a**) the case with the smallest distance between the PTV and OARs, (**b**) the case with the largest distance between the PTV and OARs, (**c**) the patient with the largest tumor motion, and (**d**) the patient with the smallest tumor motion.

As expected, the DVHs of the OARs (i.e., esophagus, heart and spinal cord) decreased at the phase of the lowest cost value compared with the phase of the highest cost value. In specific, Figure 2a and 2b show the DVHs of the cases where the overall distances between the PTV and OARs were the smallest and largest, respectively. Figure 2c and 2d demonstrate the DVHs of the cases where the motions of the GTV were the largest and smallest, respectively. In Figure 2a, the mean dose differences of esophagus, heart and spinal cord between extreme two cases were 16.63%, 18.39% and 4.48%, respectively. The mean dose differences were 6.53%, 8.80% and 5.23%, respectively in Figure 2b. In Figure 2c, the mean dose differences of esophagus, heart and spinal cord between extreme two cases were 4.75%, 15.70% and 5.36%, respectively. The mean dose differences were 5.38%, 1.82% and 2.05%, respectively in Figure 2d.

Table 3 summarizes PTV dose indices acquired from the extreme two phases for 15 patients. It shows that the average differences of the PTV mean and max doses were -0.002% and -0.03%, respectively. As shown, the PTV dose indices were very similar for the phases of extreme two cost values for all patients, implying that plan quality in PTV coverage perspective was consistent.

**Table 3:** Comparing mean/max PTV dose between the lowest cost value phase and the highest cost value phase.

Patient	Mean dose			Max dose		
	Lowest CV (Gy)	Highest CV (Gy)	Difference (%)	Lowest CV (Gy)	Highest CV (Gy)	Difference (%)
1	45.63	46.02	-0.85	46.70	47.25	-1.18
2	45.55	45.20	0.77	46.48	46.49	-0.02
3	44.80	45.15	-0.78	46.31	47.24	-2.01
4	44.86	45.10	-0.53	46.87	46.80	0.15
5	45.32	45.10	0.49	46.70	46.51	0.41
6	45.11	45.41	-0.67	46.64	46.49	0.32
7	45.07	45.07	0.00	46.68	46.49	0.41
8	45.08	45.00	0.18	46.73	46.71	0.04
9	45.24	44.95	0.64	46.51	46.11	0.86
10	45.02	44.69	0.73	46.74	46.51	0.49
11	44.43	44.98	-1.24	47.12	47.09	0.06
12	45.18	45.53	-0.77	47.11	47.08	0.06
13	45.70	45.33	0.81	47.13	47.29	-0.34
14	45.86	45.63	0.50	47.31	47.33	-0.04
15	45.28	44.99	0.64	47.29	47.13	0.34
Average	45.21±0.37	45.21±0.33	-0.002	46.82±0.31	46.83±0.39	-0.03

Abbreviations: CV = cost value.

Table 4 summarizes the EUD between the lowest cost value and the highest cost value of esophagus, heart and spinal cord. The results indicate that the EUD of the OARs significantly decreased at the phase with lowest cost value for all cases. The average EUD values for esophagus, heart and spinal cord significantly decreased by 11.95% (with *p*-value < 0.001), 9.67% (with *p*-value < 0.001) and 8.08% (with *p*-value =0.001), respectively.

**Table 4:** Comparing EUD between the lowest cost value phase and the highest cost value phase and correlation between cost value (*r*=OSD) and mean OAR dose.

Patient	Esophagus			Heart			Spinal cord			Correlation
	Lowest CV (Gy)	Highest CV (Gy)	Difference (%)	Lowest CV (Gy)	Highest CV (Gy)	Difference (%)	Lowest CV (Gy)	Highest CV (Gy)	Difference (%)	
1	27.11	30.03	-10.77	11.29	13.61	-20.55	24.90	25.02	-0.48	0.91
2	16.24	18.21	-12.13	8.77	9.39	-7.07	14.82	15.87	-7.09	0.92
3	7.41	7.74	-4.45	5.91	6.55	-10.83	7.84	7.90	-0.77	0.43
4	21.13	21.79	-3.12	7.62	7.21	5.38	11.53	13.57	-17.69	0.66
5	7.07	7.53	-6.51	8.03	8.40	-4.61	7.20	7.40	-2.78	0.30
6	16.52	19.99	-21.00	7.22	8.31	-15.10	8.67	11.31	-30.45	0.78
7	15.98	16.07	-0.56	9.48	9.59	-1.16	12.25	13.82	-12.82	0.66
8	17.62	19.33	-9.70	16.01	16.53	-3.25	7.55	7.7	-1.99	0.65
9	13.04	14.86	-13.96	9.51	9.95	-4.63	10.52	10.62	-0.95	0.95
10	13.86	15.14	-9.24	7.84	9.09	-15.94	13.25	13.75	-3.77	0.82
11	13.64	15.14	-11.00	7.29	8.90	-22.09	13.61	14.28	-4.92	0.85
12	15.15	19.77	-30.50	7.79	7.92	-1.67	8.83	9.43	-6.80	0.92
13	10.27	12.37	-20.45	6.30	7.77	-23.33	7.45	8.68	-16.51	0.92
14	13.45	15.31	-13.83	11.25	12.42	-10.40	9.58	10.54	-10.02	0.88
15	10.68	12.09	-13.20	6.44	7.75	-20.34	13.78	15.77	-14.44	0.94
Average	14.61±5.10	16.36±5.65	-11.95 (p<0.001)	8.72±2.59	9.56±2.68	-9.67 (p<0.001)	11.45±4.53	12.38±4.55	-8.08 (p=0.001)	

Abbreviations: CV = cost value.

Table 5 shows the mean doses of OARs at the phases of extreme two cost values. The mean doses of the OARs significantly decreased at the phase with the lowest cost value. The average mean doses for esophagus, heart and spinal cord significantly decreased by 10.51% (with *p*-value < 0.001), 8.67% (with *p*-value < 0.001) and 9.35% (with *p*-value < 0.001), respectively.

**Table 5:** Comparing mean OAR dose between the lowest cost value phase and the highest cost value phase and correlation between cost value (*r*=OSD) and mean OAR dose.

Patient	Esophagus			Heart			Spinal cord			Correlation
	Lowest CV (Gy)	Highest CV (Gy)	Difference (%)	Lowest CV (Gy)	Highest CV (Gy)	Difference (%)	Lowest CV (Gy)	Highest CV (Gy)	Difference (%)	
1	11.46	13.37	-16.63	8.57	10.14	-18.39	15.23	15.91	-4.48	0.94
2	9.71	10.38	-6.87	2.64	3.18	-20.32	8.47	9.41	-11.12	0.90
3	3.02	3.45	-14.24	3.83	4.47	-16.71	3.33	3.58	-7.51	0.56
4	6.24	6.62	-5.98	4.25	4.38	-3.12	5.73	6.59	-15.06	0.41
5	3.49	3.72	-6.53	8.02	8.73	-8.80	3.67	3.86	-5.23	0.79
6	9.23	11.25	-21.89	4.75	5.87	-23.64	5.53	7.19	-30.02	0.78
7	6.28	7.19	-14.40	7.90	8.10	-2.58	4.94	6.04	-22.10	0.54
8	6.26	6.48	-3.51	13.38	13.78	-2.99	3.49	3.88	-11.17	0.51
9	5.59	5.92	-5.82	8.81	9.17	-4.13	5.22	5.27	-0.98	0.83
10	9.66	10.18	-5.38	10.44	10.63	-1.82	9.29	9.48	-2.05	0.72
11	7.84	9.05	-15.43	7.02	7.13	-1.57	7.62	8.50	-11.55	0.79
12	9.41	10.02	-6.48	5.50	6.35	-15.45	3.76	3.86	-2.66	0.85
13	7.58	7.94	-4.75	6.18	7.15	-15.70	4.48	4.72	-5.36	0.78
14	8.83	9.20	-4.19	9.97	10.40	-4.31	5.92	6.23	-5.24	0.86
15	7.43	9.06	-21.94	4.14	5.05	-21.98	6.99	7.91	-13.16	0.88
Average	7.47±2.36	8.25±2.74	-10.51 (p<0.001)	7.03±2.95	7.64±2.89	-8.67 (p<0.001)	6.24±3.08	6.83±3.22	-9.35 (p<0.001)	

Abbreviations: CV = cost value.

The max doses of the OARs at the phases of extreme two cost values are summarized in Table 6. The max doses of the OARs were lower at the phase with the lowest cost value compared to the phase with the highest cost value. However, the decrease of the max dose at the phase with the lowest cost value was less than that of mean dose. The average max doses also significantly decreased for all of 3 organs (by 9.36% [with *p*-value < 0.001] for esophagus; by 4.78% [with *p*-value = 0.002] for heart; by 6.04% [with *p*-value = 0.005] for spinal cord).

**Table 6:** Comparing max OAR dose between the lowest cost value phase and the highest cost value phase and correlation between cost value (*r*=OSD) and max OAR dose.

Patient	Esophagus			Heart			Spinal cord			Correlation
	Lowest CV (Gy)	Highest CV (Gy)	Difference (%)	Lowest CV (Gy)	Highest CV (Gy)	Difference (%)	Lowest CV (Gy)	Highest CV (Gy)	Difference (%)	
1	41.72	43.11	-3.33	45.08	45.71	-1.40	40.11	40.94	-2.07	0.72
2	27.07	30.08	-11.12	44.46	44.53	-0.16	25.33	26.51	-4.66	0.86
3	13.33	13.47	-1.05	25.12	25.87	-2.99	15.48	15.34	0.90	0.53
4	31.91	32.33	-1.32	25.73	26.26	-2.06	26.69	26.93	-0.90	0.71
5	13.29	14.74	-10.91	18.49	19.25	-4.11	16.51	16.91	-2.42	0.43
6	25.34	28.72	-13.34	31.68	32.06	-1.20	16.68	21.65	-29.80	0.68
7	21.45	22.69	-5.78	27.53	29.05	-5.52	26.31	29.89	-13.61	0.80
8	26.53	27.89	-5.13	45.51	45.72	-0.46	16.73	17.29	-3.35	0.76
9	23.31	24.73	-6.09	30.74	32.53	-5.82	18.29	18.52	-1.26	0.82
10	27.46	28.91	-5.28	33.06	36.91	-11.65	44.48	44.51	-0.07	0.96
11	21.91	23.71	-8.22	39.85	43.36	-8.81	23.11	24.39	-5.54	0.67
12	24.53	30.12	-22.79	37.89	38.14	-0.66	16.34	16.71	-2.26	0.78
13	15.48	18.68	-20.67	22.72	25.91	-14.04	14.65	15.12	-3.21	0.87
14	21.88	25.28	-15.54	41.71	42.89	-2.83	17.88	20.68	-15.66	0.90
15	18.45	22.31	-20.92	31.54	36.88	-16.93	22.09	25.87	-17.11	0.93
Average	23.58±7.33	25.78±7.34	-9.36 (p<0.001)	33.41±8.65	35.01±8.49	-4.78 (p=0.002)	22.71±8.96	24.08±8.90	-6.04 (p=0.005)	

Abbreviations: CV = cost value.

## DISCUSSION

It has been shown that the OVH is an effective quality control tool for the evaluation of DVH to keep better treatment plan consistency [14, 16-18]. In this study, the OVHs at specific distance were acquired at all breathing phases, and then compared. In addition, a biological index (i.e., Tolerance Dose (TD) in this study) was included in cost value calculation to apply heavier weighting to the lower tolerance OARs. It is necessary to evaluate the feasibility of the proposed method that utilizes the cost values mainly based on overlap volume between the OARs and the expanded PTVs. Thus, we compared the correlations between cost values under various specific distances and delivered doses to OARs. Higher correlations were obtained with the cost values acquired with distance weighting than those with the cost values acquired without distance weighting in most cases except the cases with *r* = 3 cm as summarized in Table 1. As shown in Figure 1, the cost value acquired by the OSD has a higher correlation than the other cost values. Each OAR had an OSD that could acquire overlap volume between expanded PTV and the OAR in every respiratory phase; it was possible to obtain the information for the cost value of all OARs effectively without exception. Influence of delivered dose to the OAR could be increased depending on the proximity between the PTV and OARs. Therefore, it is considered that the cost value function with the OSD could acquire relatively higher correlation.

Figure 2a and 2b show the DVHs of extreme two cost value phases for two patients, one having the shortest overall distance between the PTV and OARs and the other having the longest. It was observed that the patient with the shortest distance showed more dose difference between 2 extreme cost value phases. This indicates that the surrounding OARs in the case of closer distance between the PTV and OARs can be highly affected by whether phase optimization is applied or not. Figure 2c and 2d show the DVHs of extreme two cost value cases for two patients, one with the largest GTV motion and the other with the smallest. We could observe that the mean dose differences of surrounding OARs with the largest tumor motion were greater compared to the smallest tumor motion case. Therefore, it is considered that the location of the PTV and OARs, and the magnitude of tumor motion have great influence on doses delivered to OARs at each phase. Because such factors (i.e., distance between the target and OARs and tumor motion) are different for each patient, patient-specific geometric factor should be appropriately considered for treatment planning. Consequentially, the proposed cost value function can play a role in choosing an optimal phase with minimal effort, that is, without actual plan optimization at all phases.

It is worth to note that the highest cost value was observed at a phase close to end exhalation in 67% of patients (i.e., 10 out of 15) because end exhalation is commonly used in RGRT. For those 67% of patients, dosimetric parameters of the surrounding OARs were higher at the end exhalation phase. These results basically support the results of a limited study that performed the dosimetric comparison of end exhalation and end inhalation only. For example, Cesar Della Biancia et al. reported that the average maximum dose of spinal cord decreased by 4.24% at the end inhalation phase, and this study showed that the average maximum dose of spinal cord decreased by 5.73% at the phase with the lowest cost value compared to that at the phase with the highest cost [6].

The result of this study indicates that a fair amount of patients may be treated in less optimal phase in terms of OAR sparing under current practice, and many patients can get benefit from phase optimization. Both residual tumor motion and positional reproducibility may not be same among phases. However, it is not simple to know the exact amount of them for every phase. Therefore, we did not take those factors into account in this study. Although it is ideal to completely eliminate residual motion and keep perfectly repeatable breathing cycle, more practical solution is to reduce them down to an insignificant level by using breathing coaching and/or biofeedback method so that phase-to-phase variation can be negligible in phase optimization [19-20]. In fact, various studies have been performed to improve the respiration reproducibility and reduce the residual motion. Berbeco et al. (2006) reported that the residual motion of end inhalation under breathing coaching might be equivalent to that of end exhalation [3]. In addition, Vankat et al. (2008) reported that reproducibility in displacement was improved by 55% with audio-visual biofeedback [19]. It is obvious that the phase optimization method proposed in this study can be implemented with more reliability under such techniques (i.e., breathing coaching, breath-hold and/or biofeedback) applied.

There are few limitations in this study, and we are going to address two of them considered important. First, while obtaining the cost value of lung cancer patients, both lungs were excluded. All of patients enrolled in this study satisfied that the percentage of total lung volume irradiated to > 20Gy (V20) was less than 20% in all phases, and it could be considered that the probability of lung complication would be small [21]. Therefore, we acquired cost values with the surrounding OARs only excluding the lung (i.e., esophagus, heart, and spinal cord). In fact, inhalation phases could have gotten little lower cost values if lung had been included.

Second, cost value function proposed in this study is not able to explicitly distinguish between parallel organ and serial organ. It is not an easy task to take serial organ’s importance into account separately and it would be a future project. Depending on the situation, the cost function can be easily modified to account for the situation better. For example, a certain OAR can be more important than usual (e.g., due to previous treatment and/or existing illness), and heavier weighting can be applied to such OARs. Further investigations on such situations would be beneficial and will be considered.

## MATERIALS AND METHODS

### Patients and image acquisition

With the approval of institutional review board, 15 lung cancer patients were included. Sometimes, in lung treatments, lung itself can be the most critical organ but, in this study, we focused on other OARs. When lung is of the most concern, obviously, end of inhalation, especially deep inhalation would be the first choice and there is no need of phase optimization. Therefore, we selected cases both where lung was not the dominating critical organ and tumor was located near selected OAR (esophagus, heart, and spinal cord). Table 7 presents the tumor size, tumor location, magnitude of 3D tumor motion (center-of-mass) between 2 extreme phases.

**Table 7:** PTV size, tumor location at end exhalation, and 3D tumor motion for all patients (*N* = 15).

Patient	PTV size (cc)	Tumor location	Tumor motion (cm)
1	105.76	RML	0.56
2	50.98	RML	0.43
3	46.66	RML	0.61
4	26.86	LUL	0.98
5	30.03	RML	0.47
6	51.49	LUL	0.43
7	56.56	RML	0.67
8	69.67	LLL	1.08
9	72.54	LUL	0.74
10	120.03	RML	0.41
11	60.73	RML	0.77
12	40.17	RLL	1.42
13	50.27	LLL	1.78
14	39.63	LUL	0.63
15	50.776	RML	0.74

Abbreviations: RML = right middle lobe; RLL = right lower lobe; LLL = left lower lobe; LUL = left upper lobe.

4DCT images for all of the patients were obtained using a multi-slice CT scanner with 0.3 cm slice thickness (SOMATOM Sensation 16; Siemens Medical Solutions, Erlangen, Germany) and a motion-monitoring system (AZ-733 V; Anzai Medical, Tokyo, Japan) which utilized a pressure sensor, fixed in the upper abdominal region with an elastic belt, to detect respiratory motion signal. The projections were retrospectively sorted into ten respiratory phase bins equally distributed over the breathing cycle using the Syngo software package (Siemens Medical Solutions). During the acquisition of 4DCT images, the patients were advised to breathe freely and regularly. However, neither abdominal compression nor real-time coaching was given.

### Contour delineation and treatment planning

For all patients and all phases, structure delineation (i.e., defining the target volume and OARs - esophagus, heart and spinal cord) was performed using an Eclipse treatment planning system (Version 10.0, Varian Medical System, Palo Alto, CA). To avoid inter-observer variation, one observer took the charge of delineation for all of 15 patients. The GTV of each phase was defined under the same lung window setting. The PTV of each phase was defined as a 5 mm expansion beyond the GTV. The contour of esophagus was performed on outer esophageal wall from its most cranial appearance to the esophageal hiatus [11-12]. The heart was defined with outer peri-cardial sac from the level of the inferior aspect of the pulmonary artery to the apex of the heart [22]. Spinal cord was contoured over the complete superior-inferior direction.

Various factors such as beam energy, wedge field, number of beams and beam orientation were equally applied to every phase of the patients to keep consistency in treatment planning. However, the field size of each phase varied depending on the PTV location and beam’s eye view. 3D conformal radiation therapy (CRT) plans with 12 beams were generated using a 6 MV photon beam. The prescription dose chosen in this simulation study was 45 Gy delivered in 25 fractions with the 95% coverage of the PTV.

Although the total dose and fraction size were not necessarily same for every patient in actual treatment, the same prescription dose was applied for effective analysis in this study, where the main goal was to investigate the correlation between delivered dose to OARs and cost value calculated using the geometrical relationship and the biological effect [23].

### Planning phase optimization

#### A. Cost value function

It is not easy to define a distance relationship between the PTV and OARs in thoracic region where there are multiple critical structures such as esophagus, heart and spinal cord. The OVH is able to quantify the three dimensional geometric relationship between the PTV and OARs. The OVH(*r*) represents a 1-dimensional distribution that describes each OAR’s fractional volume overlapping with uniform PTV expansion by a specific distance *r* [13, 16].

**Figure 3:**
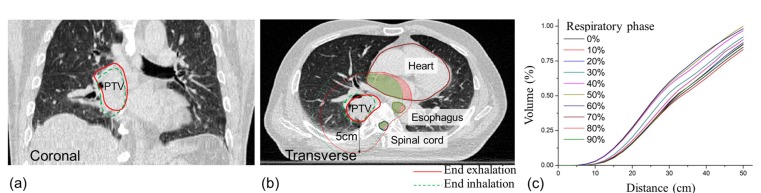
Illustration of the changes of geometric relationships between the PTV and OARs (esophagus, heart and spinal cord) in different respiratory phases (**a**) phase–wise PTVs in a coronal CT image, (**b**) phase-wise expanded PTVs and OARs in an axial CT image, and (**c**) example of OVH curves for esophagus.

Because the geometric relationship between the PTV and the surrounding OARs may differ from phase to phase due to respiratory motion, we used the OVH as a descriptor to quantify each OAR volume overlapped with the expanded PTV at each phase. Figure 3 shows an example where geometrical relationships between the PTV and OARs changed from respiratory phase to phase (end of inhalation to end of exhalation in this illustration). If a phase shows a larger overlap volume for a considered OAR when the same specific distance is applied, it is reasonable to assume that planning at that phase is difficult to spare the OAR.

To compare the relative geometric relationship between each breathing phase, the OVHs of each breathing phase were normalized to the largest of the PTV expansion distances. Because the distances between the PTV and surrounding OARs were typically shorter than 5 cm for our patient population, and also it was relatively easy to spare OARs that were located farther than 5 cm from the target, the maximum expansion distance was chosen to be 5 cm in this study. Expansion distances for OVH calculation ranged from 0.25 cm to 5 cm at 0.25 cm interval. Treatment planning using biological parameters such as tumor control probability (TCP), normal tissue complication probability (NTCP) and EUD has been performed to reduce radiation-induced toxicity for normal tissues [24-25]. While, however, utilization of such parameters (i.e., TCP, NTCP and EUD) is not fully mature, TD_50_ (the tolerance dose for a 50% complication) is universally used in current practice. In this study, therefore, we propose to utilize the OVH and TD_50_ of Emami-Burman parameter to establish a cost value function for breathing phase optimization [26]. This cost function, in detail, is defined as:

$$Cost\;Value=\frac{\sum_{i}^{I}\left({OVH}_{i}\left(r\right)\times \left(1/TD_{i}50\right)\times W_{i,d}\right)}{\sum_{i}^{I}\left(\left(1/TD_{i}50\right)\times W_{i,d}\right)}$$ … (1)

Where, OVH_*i*_(*r*) is the percentage of fractional volume of *i-*th OAR within a specified distance *r*, TD_*i*_50 is the tolerance dose to *i-*th OAR with which NTCP is 50%, and W_*i,d*_ is the weighting factor that takes into account the proximity between the PTV and OARs (distance weighting). Because dose decreases with the distance from the target, it is reasonable to put heavier weighting on overlap volumes located closer to the target than those placed farther, and W_*i,d*_ reflects dose reduction according to the distance from the PTV. To obtain W_*i,d*_, the average trend line of dose profile between the edge of the PTV and the edge of the expanded PTV (5 cm) for 3 patients (#1, 2, and 3) was utilized. In addition, in order to evaluate the effect of distance weighting on cost value function, cost values without distance weighting (i.e., W_*i,d*_ = 1) were acquired. Intuitively, planning at that phase with the lowest cost value would bring an optimized dose distribution for the OARs saving.

#### B. Relationship between cost value and specific distance ‘*r*’

It is important to choose a proper specific distance ‘*r*’ within which important OARs exist and doses to them are significant enough. Having this in mind, we evaluated cost values with 4 different specific distances, 3 cm, 4 cm, 5 cm and OSD. The OSD for an OAR was defined as the shortest distance at which the expanded PTVs and the OAR overlap in every respiratory phase. Thus, each OAR has its own OSD and ‘*r*’ in equation (1) for *i*-th OAR can be expressed as OSD_*i*_. After cost values for all phases were acquired under various specific distances (i.e., 3 cm, 4 cm, 5 cm and OSD), Pearson correlation between the cost values and the summation of equivalent uniform dose (EUD) over considered OARs was evaluated.

EUD was obtained using:

$${EUD}_{Sum}=\sum_{i=1}\left({\left(\sum_{j=1}\left(v_{ij}D_{ij}^{ai}\right)\right)}^{\frac{1}{a_{i}}}\right)$$ … (2)

Where ‘a_*i*_’ is a model specific parameter of *i*-th organ, and v_*ij*_ represents the *j*-th partial volume of *i*-th organ receiving dose D_*ij*_ in Gy.

### Dosimetric comparison

For all of 15 patients, both the highest cost value phase and the lowest cost value phase under the specific distance chosen to be the most effective in the previous section were identified. The DVHs of the phases of extreme two cost values were compared, and the mean/max doses of PTV, esophagus, heart, and spinal cord were analyzed. The paired t-test was used to compare the dosimetric differences between phases of the extreme two cost values. A *p*-value < 0.05 was considered to be significantly different. All analyses were performed using Statistical Package of Social Sciences, version 12.0 (SPSS, Chicago, IL, USA).
